# The complexities of measuring access to parks and physical activity sites in New York City: a quantitative and qualitative approach

**DOI:** 10.1186/1476-072X-8-34

**Published:** 2009-06-22

**Authors:** Andrew R Maroko, Juliana A Maantay, Nancy L Sohler, Kristen L Grady, Peter S Arno

**Affiliations:** 1 Department of Environmental, Geographic, and Geological Sciences, Lehman College of the City University of New York, Bronx, USA; 2 Earth and Environmental Sciences, The Graduate Center of the City University of New York, New York, USA; 3 Community Health and Social Medicine, Sophie Davis School of Biomedical Education of the City University of New York, New York, USA; 4 Department of Health Policy and Management, School of Public Health, New York Medical College, Valhalla, USA

## Abstract

**Background:**

Proximity to parks and physical activity sites has been linked to an increase in active behaviors, and positive impacts on health outcomes such as lower rates of cardiovascular disease, diabetes, and obesity. Since populations with a low socio-economic status as well as racial and ethnic minorities tend to experience worse health outcomes in the USA, access to parks and physical activity sites may be an environmental justice issue. Geographic Information systems were used to conduct quantitative and qualitative analyses of park accessibility in New York City, which included kernel density estimation, ordinary least squares (global) regression, geographically weighted (local) regression, and longitudinal case studies, consisting of field work and archival research. Accessibility was measured by both density of park acreage and density of physical activity sites. Independent variables included percent non-Hispanic black, percent Hispanic, percent below poverty, percent of adults without high school diploma, percent with limited English-speaking ability, and population density.

**Results:**

The ordinary least squares linear regression found weak relationships in both the park acreage density and the physical activity site density models (R_a_^2 ^= .11 and .23, respectively; AIC = 7162 and 3529, respectively). Geographically weighted regression, however, suggested spatial non-stationarity in both models, indicating disparities in accessibility that vary over space with respect to magnitude and directionality of the relationships (AIC = 2014 and -1241, respectively). The qualitative analysis supported the findings of the local regression, confirming that although there is a geographically inequitable distribution of park space and physical activity sites, it is not globally predicted by race, ethnicity, or socio-economic status.

**Conclusion:**

The combination of quantitative and qualitative analyses demonstrated the complexity of the issues around racial and ethnic disparities in park access. They revealed trends that may not have been otherwise detectable, such as the spatially inconsistent relationship between physical activity site density and socio-demographics. In order to establish a more stable global model, a number of additional factors, variables, and methods might be used to quantify park accessibility, such as network analysis of proximity, perception of accessibility and usability, and additional park quality characteristics. Accurate measurement of park accessibility can therefore be important in showing the links between opportunities for active behavior and beneficial health outcomes.

## Introduction

Environmental justice is the fair and equitable distribution of both the environmental "bads," such as hazardous waste sites, and the environmental "goods," such as parks, open space, and recreational opportunities. For more than a decade, Geographic Information Systems (GIS) have been used to examine the spatial realities of environmental justice [1-11]. GIS methods have been applied in environmental justice research primarily in the analysis of the spatial relationships between sources of pollution burdens and the characteristics of potentially affected populations. Environmental justice research has therefore focused on analyzing the disproportionate exposure of pollution on communities comprised of vulnerable groups, such as racial/ethnic minorities and socio-economically disadvantaged groups, and the concomitant effects of this pattern on health and environmental disparities [12-14]. GIS has been less often used to analyze the relationship between socio-demographic and environmental "goods," such as health-promoting land uses and positive aspects of the built environment.

Previous studies have documented that proximity to parks and open spaces has a positive influence on engaging in active behaviors, like walking and running for exercise [15-19]. Other studies have analyzed how the availability of outdoor space impacts on specific health outcomes, like community-level rates of mortality, cardiovascular disease, diabetes, and obesity [20-26]. The underlying hypothesis is that since individual-level risk factors for these highly prevalent diseases do not fully explain disparities in their distribution across population groups, or even disparities across population groups in health behaviors that are related to these diseases, modifiable environmental factors may help us to develop fuller models explaining health disparities in these health outcomes and related health behaviors. This research is of interest to public health and policy analysts who are developing interventions and policies that can mitigate health disparities that persist across socio-economic groups in the USA.

If environmental factors help us to understand the distribution of health outcomes in the population, then one might expect that active outdoor space would be less available to populations with overall worse health outcomes. Since low SES populations and racial/ethnic minorities experience worse health outcomes in the USA [27,28], access to parks and physical activity sites becomes an environmental justice issue. However, research findings have been contradictory, which suggests a complex relationship among socio-demographics, outdoor space, and individual-level health factors.

Many researchers have endeavored to evaluate access to parks and recreational facilities, and have used various methods and measures to do so. However, there are many pitfalls in developing a measure or index of accessibility, and even the more sophisticated analyses have some problems in matching their indices with reality. Research on this topic has grown more nuanced in recent years, but many of these analyses still present some methodological difficulties, which may call into question their findings, especially considering that many of these studies show significantly contradictory results (see table 1).

**Table 1:** Summary of selected park accessibility research.

**Research Study**	**Study Area(s)/Unit of Analysis/Independent Variables**	**Measuring**	**Methods**	**Findings**
*Abercrombie et al, 2008* [31]	Study Area: Metro Baltimore/DC area (MD) Unit: census block groups Independent Variables: % minority, median income, pop size, geographic size, and % pop < 18.	Number of private rec. facilities and public parks per block group; size of rec. space. Number of parks and facilities were recoded into categories based on # per block group and the park size was divided into four categories based on Mertes and Hall's classification system.	Neighborhoods selected by variation in walkability and median income. Socio-demographic variables in tertiles (low, medium, and high). Two way analysis of covariance: # private facilities, # parks, & largest park size across block groups.	No signif. effect of income or % minority on # private rec. Mixed-race neighborhoods had highest number of parks, regardless of income. Low- and middle-income pop. in white block groups and high-income groups in minority block groups had lowest park access.

*Estabrooks et al, 2003* [29]	Study Area: small American Midwestern city (not specified) Unit: census tracts Independent Variables: % unemployed, per capita income; % pop. below poverty threshold, education (less than h.s. diploma). Racial/ethnic characteristics	Availability of PA resources and accessibility as pay-for-use and free-for-use. Raw counts of numbers of PA facilities per census tract.	Multivariate analyses of variance of PA resource availability and accessibility by neighborhood SES; Univariate analyses of variance to determine whether income differed on the number of pay-per-use and free-for-use facilities.	Low- and medium-SES neighborhoods have signif. fewer PA resources than high-SES neighborhoods. Low- and medium-SES neighborhoods have signif. fewer free-for-use resources than high-SES neighborhoods

*Moore et al, 2008* [39]	Study Areas: Forsyth County, NC; Manhattan & Bronx, NY; Baltimore City & County, MD Unit: census tracts, blocks, and 100-meter grid cells (kernel density) Independent Variables: total pop, racial/ethnic pop, land area, median household income.	Presence of resources, as well as densities & types of resources. Public-use parks, commercial and public rec. The total number of resources obtained by summing the resources at each location, weighted by the count when appropriate.	Kernel density of recreational resources, weighted by # of resources and types; binomial regression for probability of having access as function of SES and demographic factors.	Minority & low income areas signif. less likely to have fee-for-use rec. Densities of public rec. within parks were signif. higher in minority and low-income tracts, even after adjustment for pop.

*Nicholls, 2001* [34]	Study Area: Bryan, TX Unit: census tracts Independent Variables: Pop density, % non-White; % black; % Hispanic; % < 18; % > 64; % renter occupied housing units; mean housing value; mean rent	Equity and accessibility to parks: ease with which a site can be reached and fairness of distribution of parks.	Buffering Euclidean & street network distance for accessibility; comparison of pop. factors of areas w/good access to pop. factors in areas w/o good access	Large areas of the city are not within 1/2 mile of a park access point, by either the straight-line or network distance. < 40% of pop has good access. All pops seem equally well-served by the parks, and the parks are well-distributed amongst less advantaged groups

*Talen, 1997* [32]	Study Areas: Pueblo, CO, and Macon, GA Unit: census blocks Independent Variables: % non-white (Macon); % Hispanic (Pueblo); % < 18 years; vacant units; owner occupied units; Median housing value; % housing units w/> 1 person per room; % households w/no spouse present	The spatial clustering of park access scores with the spatial clustering of SES variables. Also used a measure of accessibility at the census block level based on amounts of park acreage within certain distances of residential areas.	Access measure consists of the total amount of park acreage located within a specified travel distance between each block and each park, using street network distance between centroids of blocks and centroids of parks.	Spatial autocorrelation for both cities is significant for park access measures. Park access in Pueblo favors higher-income areas. In Macon, access to parks tends to favor lower-income areas.

*Talen and Anselin, 1998* [35]	Study Area: Tulsa, OK Unit: census tracts Independent Variables: % pop < 18; % non-white; median housing value	Spatial distribution of playgrounds using the shortest path distances over street network from census tract centroids.	Compares the results of "container method" w/the geographic access measures obtained by gravity model (travel cost measure).	The playgrounds are not distributed evenly throughout the city, but are also not predicted by any specific socio-demographic variables.

*Timpiero et al, 2007* [30]	Study Area: Melbourne, AU Unit: postal districts Independent Variables: Index of relative SES disadvantage (income, education, occupation, family composition, dwelling structure).	Density and area of various categories of open space in relation to SES within each postal district.	container approach was used correlating numerous variables (# of OS facilities, OS area, OS density, etc) to SES index	Greater # of o.s in lowest SES districts; once normalized by pop, differences not signif.

*Wolch et al, 2005* [33]	Study Area: Los Angeles, CA Unit: census tracts Independent Variables: Total pop; racial/ethnic pop; pop < 18; median household income; % persons in poverty.	Park access = park acres/1,000 pop (total pop and < 18 pop); % of tract pop (total and < 18) within 1/4 mile of a park boundary; Park acres/1,000 pop (total and < 18) living within the 1/4 mile buffer.	1/4 mile buffers around parks creating accessible park acreage per census tract. Estimates of total area within a 1/4 mile of park and total accessible population per tract were calculated.	Low-income and concentrated poverty areas have relatively low levels of park resources and accessibility. African American,, Latino, and Asian American pops have low rates of park access compared to white-dominated areas.

Abbreviations used in table: o.s. = open space; rec. = recreational facilities; pop = population(s); PA = Physical Activity; SES = socio-economic status

One of the most common methods used in examining access to park space is called the "container approach." This approach measures access by determining whether or not there is a park or recreational facility within a particular geographic unit of aggregation (e.g., zip code, census tract, or neighborhood), rather than using or developing an actual proximity measure such as Estabrooks, et al, 2003 [29]. In this container approach, the number of parks per areal unit is then summed and associations between this count and various population characteristics, such as SES, can be estimated for the chosen unit of aggregation.

**Figure 1:**
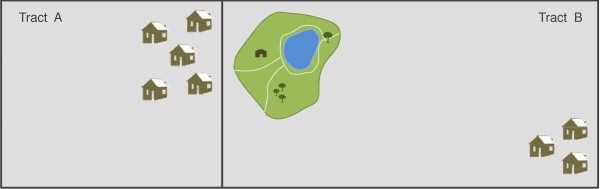
**Problems with the "container approach**. "In Tract A, population lives in close proximity to a park, but the container approach would report "no access," because the park is in a different enumeration unit. In Tract B, the population lives far from the park but the container approach would report "access," because the park is in the same enumeration unit.

This may be problematic in arriving at an accurate depiction of park access. For example, a person may live directly next to a park, but if the park is located in a different unit of aggregation (e.g., zip code, census tract, etc.), it will not be counted as "accessible" for that person. Additionally, populations that are distributed heterogeneously across a large areal unit may also introduce error in estimating park access. A population that is distributed heterogeneously within the areal unit may also introduce error in estimating park access, since, especially with larger areal units, the population may be concentrated in portions of the geographic unit that are not in close proximity to the park, although still within the same unit (see figure 1).

A number of previous studies have used this "container" method for evaluating whether or not a person has good access to parks [29-31], and have found contradictory results when correlated with neighborhood SES (lack of access to parks positively correlated with low SES versus negatively correlated with low SES). These inconsistent results may be due to the container method itself. In addition to the boundary problem discussed above, this method is problematic in that it often does not take into account the underlying population structure and density of the areal unit, or the relative size of these areal units. Thus it is not a fair comparison since areal units with larger populations require more parks than an equivalent areal unit with fewer people in order to be equitable.

Additionally, although some of these studies use actual park acres per geographic unit in their calculations, others simply count the number of parks and facilities per geographic unit. However, creating a simple count of parks and basing equity analysis on that count does not consider the actual amount of park space available to residents, since one park may be substantially smaller than another and therefore should not receive an equal weight in the calculations.

Other studies have used proximity analysis based on "walkability" distances, which is a more refined measure of access, by setting certain distances to parks as a proxy for access, such as 1/4 mile (~400 m) or 1/2 mile (~800 m) as a standard walking distance [32,33]. However, access by proximity or distance often does not take into account the actual street network, merely Euclidean distance.

There have been a few studies using the street network to calculate distance to parks, for instance, Nicholls (2001) and Talen and Anselin (1998) each of whom compared straight-line distance with street network distance [34,35]. The Nicholls study found that approximately 80% of the area studied (Bryan, Texas) were not within 1/2 mile (~800 m) of any park (as measured by network distance), but that the less affluent neighborhoods tended to be better served by parks than the more affluent areas. In the Talen and Anselin study, the distribution of playgrounds in Tulsa, OK, as measured by various methods including the network analysis, could be considered "unpatterned inequality." The playgrounds were not distributed evenly throughout the city, but were also not predicted by any specific socio-demographic variables.

Kernel density estimation, or kernel smoothing, is another method for measuring accessibility. "Kernel density estimation involves placing a symmetrical surface over each point, evaluating the distance from the point to a reference location based on a mathematical function, and summing the value of all the surfaces for that reference location. This procedure is repeated for all reference locations." [36] Kernel density estimation creates a statistical surface so that, for instance, there is an accessibility value as measured by park density, mapped at every point in the study area.

Kernel density estimation is typically considered a more refined spatial statistical model than the container approach. It can give an estimation of accessibility for every point in the study area, not just a binary answer of "within walking distance" or "not within walking distance," as in both the fixed-distance proximity and the network analyses. There have been very few studies of park access using the kernel density method, although this method has been used extensively in other types of analyses [37,38]. Moore, et al. (2008) used the kernel density estimation method to compare park access in three USA locations, and found that although pay-for-use recreational venues were more likely to be located in white and more affluent neighborhoods, public parks tended to be more equitably distributed, and densities of recreational facilities within parks were significantly higher in minority and low-income census tracts than in white and higher-income tracts, even after adjustment for population [39].

## Methodology

This analysis uses the kernel density estimation approach to test whether access to park space is associated with neighborhood race/ethnic composition and SES in New York City. Densities of both park acreage and physical activity sites are mapped and correlated with SES measures. The two main categories of data used in this analysis were park information and socio-demographic information.

### Data – parks

**Figure 2:**
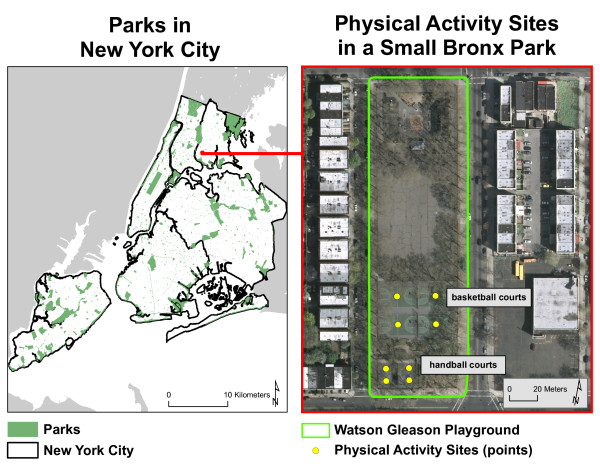
**Parks in NYC and physical activity sites in Watson Gleason Playground, Bronx, NY**. This example demonstrates that the physical activity sites in a relatively small park are not homogeneously distributed within the park, and this tends to be even more pronounced in larger parks, therefore affecting accessibility. Data Sources: NYC Dept. of Parks and Recreation collaboration with Lehman College "Geographic Features Identification Project," 2006; Orthophoto: NYCMap NYC Dept. of Information Technology and Telecommunications, 2002.

The park extent data was created by the New York City Department of Parks and Recreation and represents all land owned by the Parks Department as polygons (see figure 2). These polygons are coded into various classes such as green streets, small parks, and large parks.

The park features data (elements within the parks) were created through a collaboration between the New York City Department of Parks and Recreation and Lehman College of the City University of New York. Researchers traveled to all of the New York City parks carrying portable GPS units and recorded the locations of many of the parks' features, including items such as drinking fountains, comfort stations (rest rooms), flag poles, stairways, historical markers, statues, beaches, courts, ball fields, and other recreational areas (see figure 2). This point data (latitude and longitude) were rectified with aerial photos and further processed into a more accurate and useable dataset.

**Figure 3:**
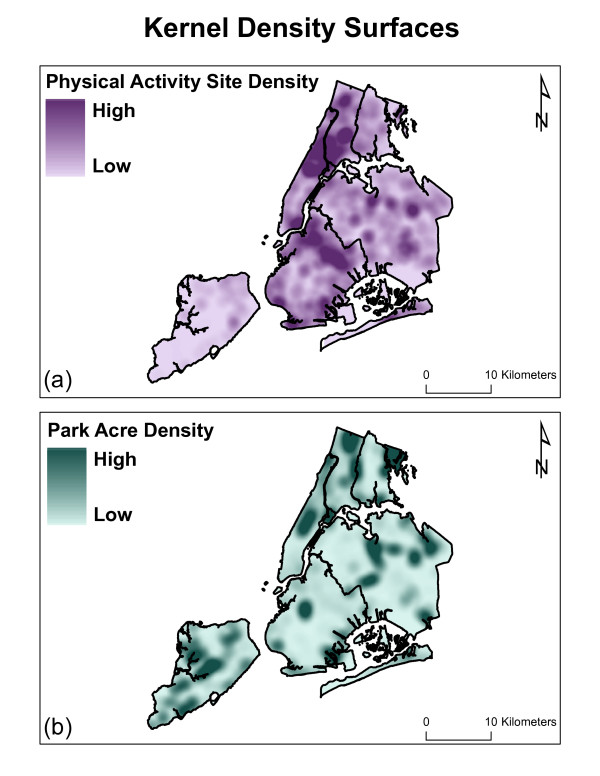
**a & b: Kernel Density Surfaces**.

For this study, two separate data layers were created based on the park information. The first layer was designed to represent park area and the second to represent physical activity sites. The park area layer was created by first identifying each acre of New York City as either 'park' or 'no park'. If there is any park space in any given acre, the pixel representing that acre was given a value of '1' ('park'). Otherwise, that pixel would have been given a value of '0' ('no park'). This grid was then converted into a statistical surface using the kernel density technique. This method involves a kernel function, which uses a moving window to apply differential weights to objects based on proximity. Thus objects that are close are weighted more heavily than more distant objects. Based on a sensitivity analysis, the bandwidth parameter for the kernel function was assigned a 1.6 km (1 mile) radius. This sensitivity analysis tested three different kernel sizes (1/4 mile, 1/2 mile, and 1 mile). Empirically, the one mile kernel bandwidth explained more of the variance in the model than the other bandwidths. This distance was also determined to be an appropriate kernel bandwidth for defining a feasible walking distance for park accessibility based on other research [39]. The kernel density estimation resulted in a 50 meter raster surface representing the density of park acreage for New York City (see figure 3a). This surface is used as a proxy for accessibility.

To prepare the data layer for physical activity sites, each discrete non-linear park feature that was identified as activity promoting (i.e. something that encourages caloric expenditure) was extracted from the main parks database, converted to a point (if necessary) and given a value of '1' (1 = physical activity site). The features that were coded are: basketball courts, handball courts, tennis courts, volleyball courts, multipurpose courts, soccer fields, baseball fields, football fields, swimming pools, hockey rinks, golf courses, and running tracks. Kernel density estimation was again performed, this time in order to estimate the density for physical activity sites in New York City. A 1.6 km radius was used as the bandwidth and a 50 meter resolution raster surface was created (see figure 3b).

These two density surfaces were used as proxies for access to park space (acres) and active recreation (physical activity sites), following the assumption that where there are higher densities of resources, access is greater.

### Data – Socio-demographics

**Figure 4:**
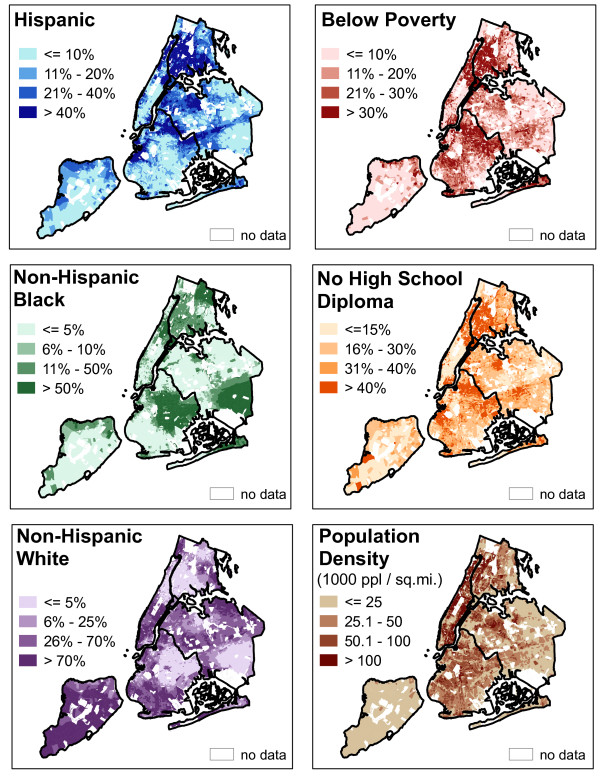
**Socio-demographics in NYC**. The maps show the SES variables used in the models. Data Sources: U.S. Bureau of the Census, 2000.

In order to evaluate the possibility of unequal access to these park measures based on socio-demographic characteristics of the population, information was gathered from summary file 1 (SF1) and summary file 3 (SF3) of the 2000 USA census at the block group level. The measures included in this analysis were percent non-Hispanic black, percent Hispanic, percent of adults aged 25 years and older with no high school diploma, percent below poverty, percent who do not speak English well or do not speak it at all, and population density (see figure 4).

### Joining parks data with demographic data

To link the raster park data with the vector socio-demographic data, zonal statistics were used within the spatial analyst extension of ArcGIS. This process aggregates and statistically summarizes the values of the raster cells whose centroids fall within the corresponding block group. As a result, each census block group in New York City is given an average value for the park acre density and physical activity site density of the grid cells that fall within its boundaries. This aggregates the kernel density-derived statistical surfaces (acres and physical activity sites) to the same geographic unit as the socio-demographic data.

## Quantitative analysis/results

Two discrete statistical methods were used on the data: ordinary least squares linear regression (OLS) and geographically weighted regression (GWR). The datasets used in the analyses were identical. Census block groups with fewer than 256 residents were excluded (lowest 5%) in order to stabilize the model. Block groups that had missing data from any of the variables were also excluded (~ 0.1%). After the data were cleaned and prepared, 5,439 block groups out of the original 5,732 remained (94.9%). Log10 transformed park acreage density (ACRE) and log10 transformed physical activity site density (PAS) were used as the dependent variables. The independent variables included: percent non-Hispanic black, percent Hispanic, percent of adults with no high school diploma, percent below poverty, percent with limited English language ability, and population density.

When analyzed globally (with OLS) it appears that PAS and ACRE behave differently with respect to the independent variables (see table 2). There are some interesting changes in directionality with the percent non-Hispanic black and the percent limited English language variables when comparing PAS and ACRE scores. Both variables are positively associated with the density of physical activity sites yet negatively associated with park acre density. Since both models explain a relatively small amount of the variance in the dependent variables (23% and 11% for PAS and ACRE, respectively), the same models were recreated using a geographically weighted regression (GWR) in an attempt to account for potential spatial non-stationarity (i.e. local variation in the relationships).

**Table 2:** OLS Regression t-values.

		**t-values**					
		
**MODEL**	**R**^2^	**% non-Hispanic black**	**% Hispanic**	**% no high school**	**% below poverty**	**population density**	**% limited English language**
**PAS**	.231	13.5**	3.1**	-0.7	13.7**	17.4**	2.2*
**ACRE**	.114	-3.7**	16.8**	-7.0**	5.5**	12.6**	-12.6**

* = p < .05, ** = p < .005.

Geographically Weighted Regression (GWR) is a technique developed by Fotheringham, Brunsdon, and Charlton designed to quantify locally varying relationships among data, rather than the more common global relationships (e.g., OLS regression). These local relationships may vary over space therefore accounting for any potential spatial non-stationarity. In other words, the measurement of the relationship may be partially dependent upon where the measurement is taken. Fotheringham, et al (2002) suggest several reasons for locally varying relationships, such as sampling variation, a misspecification of the model (e.g., omitted variables or those which are not measureable), or simply a relationship which intrinsically varies over space [40]. GWR is essentially a modification of traditional regression techniques, except rather than calculating global parameter estimates based on one regression, GWR performs many local regressions, each of which is influenced by the surrounding data. In this way, GWR shows local variations in the relationships and accounts for spatial non-stationarity. In this study, we used GWR to enable us to see where these relationships vary and hypothesize as to why they behave as such. By definition, the R^2 ^will rise and the models will technically perform better. An adaptive kernel, which attempts to minimize the Aikaike Information Criterion (AIC) by running many iterations of the model, was used to determine the optimal number of nearest neighbors for the regressions. This adaptive kernel, rather than a fixed bandwidth, was chosen in order to reduce edge effect since we do not have detailed park data for the areas outside New York City. Although this method ensures a sufficient local sample size for all regression points, it can result in unstandardized geographic sizes for individual regression points (particularly near study boundary edges) which can lead to overly smoothed results for those areas. Although this is certainly a limitation, it may not be critical in this study since we were interested in examining the potential environmental justice issues regarding New York City residents and New York City parks only, that by necessity must be confined to the boundaries of the city's jurisdiction. The adaptive kernel method resulted in the utilization of 271 nearest neighbors for the PAS model and 279 nearest neighbors for the ACRE model. Although these samples are certainly large enough for a stable model, they represent only approximately 5% of the original data, suggesting that the relationships are quite local (as the number of nearest neighbors used in the GWR approaches the total number of observations in the data, the model becomes more similar to a global OLS). The local nature of these relationships is further confirmed by a Monte Carlo test for spatial variability, which was executed within the GWR3 software where the spatial variability of all the parameters, with the exception of percent of adults without a high school diploma in the PAS model, were shown to be significant (see tables 3 and 4). The adjusted R^2 ^values were .70 for the PAS model, and .68 for the ACRE model. The Aikaike Information Criterion (AIC) was lower for the GWR models when compared with the global (OLS) models, suggesting that the former perform better than the latter. Model parameter summaries are provided in tables 2 and 3. These data show the dynamic nature of the parameters through their range of values which often switch signs after the first quartile. This, once again, suggests a non-stationary relationship.

**Table 3:** PAS GWR model parameter summaries.

**Parameter**	**Minimum**	**1^st ^Quartile**	**Median**	**3^rd ^Quartile**	**Maximum**
% non-Hispanic black*	-0.0316	-0.0008	0.0018	0.0049	0.0608
% Hispanic*	-0.0602	-0.0017	0.0016	0.0043	0.0322
% below poverty*	-0.0108	-0.0018	0.0006	0.0022	0.0144
% with no high school diploma	-0.0148	-0.0017	0.0003	0.0020	0.0146
% limited English language*	-4.1154	-0.3686	-0.0484	0.2927	2.8757
population density*	-0.0037	-0.0003	0.0000	0.0006	0.0152

* = spatial variability p value < .01

**Table 4:** ACRE GWR model parameter summaries.

**Parameter**	**Minimum**	**1^st ^Quartile**	**Median**	**3^rd ^Quartile**	**Maximum**
% non-Hispanic black*	-0.0670	-0.0044	0.0000	0.0047	0.0892
% Hispanic*	-0.0378	-0.0037	0.0013	0.0062	0.0372
% below poverty*	-0.0311	-0.0022	0.0007	0.0040	0.0185
% with no high school diploma*	-0.0435	-0.0047	-0.0013	0.0014	0.0269
% limited English language*	-6.3580	-0.8106	-0.2735	0.3225	5.8639
population density*	-0.0121	-0.0012	-0.0001	0.0004	0.0049

* = spatial variability p value < .01

**Figure 5:**
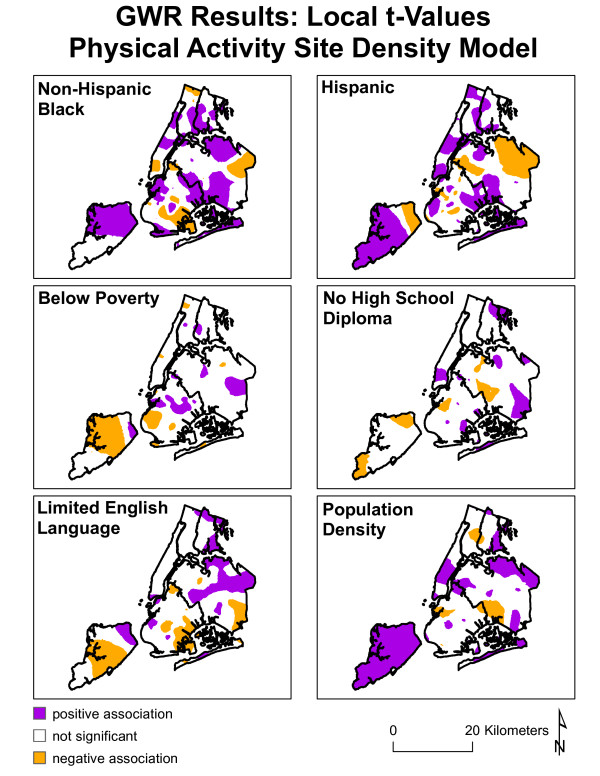
**Geographically Weighted Regression of Physical Activity Sites**. Spatial distribution of local t-values from PAS GWR linear regression. Purple areas suggest positive association between physical activity site density and the independent variable, white areas suggest no statistically significant relationship, and gold areas suggest negative associations.

**Figure 6:**
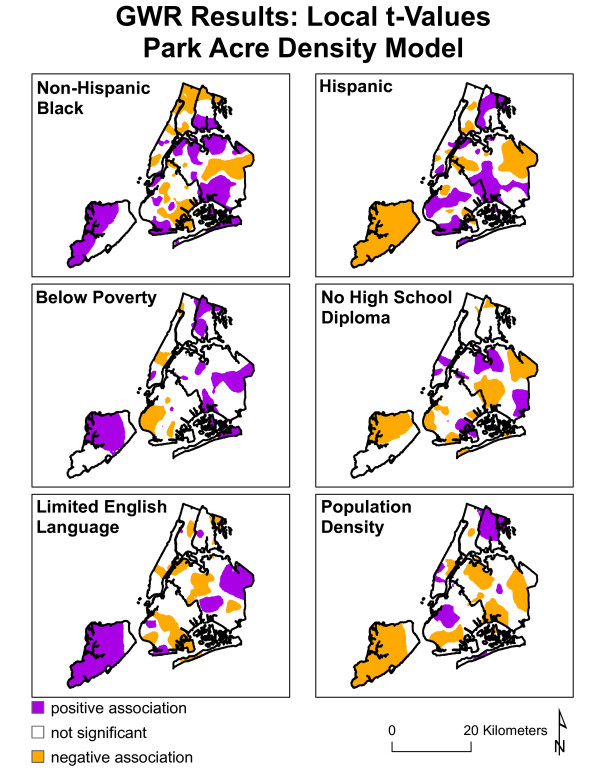
**Geographically Weighted Regression of Park Acreage Density.** Spatial distribution of local t-values from park acre density GWR linear regression. Purple areas suggest positive association between park acre density and the independent variable, white areas suggest no statistically significant relationship, and gold areas suggest negative associations.

Since GWR allows the relationships to fluctuate, it can be difficult to summarize or conceptualize concisely via tables or graphs. As such, maps have been created that illustrate the variability of the relationships between the independent and dependent variables for both models (see figures 5 and 6). The maps depict the directionality of t-values of the parameters as calculated by the GWR. 'White spaces' are areas that do not have a statistically significant relationship. The purple areas have a positive association between the variable in question (e.g. percent non-Hispanic black) and the dependent variable (e.g. PAS). It is important to note that this describes the directionality of the relationship, not the presence or absence of any single variable. For instance, one could have a significant positive relationship in areas that have a high percentage of non-Hispanic black residents and high ACRE values, or areas that have a low percentage of non-Hispanic black residents and low ACRE values (while adjusting for the other variables). Areas with statistically significant negative relationships, again with regard to the directionality of the associations, are depicted by the orange/gold color. These areas suggest that when the independent variable (e.g. percent non-Hispanic black) is high the dependent variable (e.g. ACRE) would be low while adjusting for the other independent variables, and vice versa.

The GWR analysis identified the relationships between park access measures and socio-demographic variables as behaving inconsistently across New York City. These idiosyncrasies could benefit from a qualitative evaluation of the relationship between SES characteristics with physical activity sites and park density.

## Qualitative analysis and results

### Justifications for qualitative analysis

The GWR analysis revealed a tendency toward what has been termed "unpatterned inequality," meaning that while the parks and physical activity sites are not evenly distributed across the city in a geographical sense, neither are they predicted globally by race/ethnicity, income or the other variables typically investigated in environmental justice analyses. There are no discernable consistent associations between park access and socio-demographic indicators [35]. This is, of course, not to say that everyone has equal access to parks and physical activity sites, or that all neighborhoods have good access to these resources. Certainly, a quick glance of a map of New York City's parks indicates that not all parts of the city are equally well-served by parks.

Therefore, we thought it would be beneficial to investigate case study areas on a more detailed basis, which might illuminate the spatial incongruities that exist. More explanatory power may be realized through a qualitative analysis, which includes historical background of the parks and surrounding neighborhoods, a description of the past and current socio-demographics, and an overview of the physical aspects of the study areas.

New York City has a complex relationship between its physical infrastructure and its population distribution. Many parks are quite old and were established in neighborhoods having very different socio-demographic characteristics than those of the same neighborhoods today. Parks are a special category of fixed infrastructure for that reason – most occupy large parcels of land and once they are established, it is unlikely that they will be eliminated or moved. The populations surrounding them, however, are quite changeable. This is why it is difficult to ascribe environmental justice implications to the locations of parks. It is still instructive to try to determine which populations, if any, are currently underserved by parks and recreational spaces in New York City, despite the original purpose of the parks, and who those parks were intended to serve.

### Selection of study areas/delineation of study area boundaries

Our objective in the qualitative analysis was to compare two case study areas that behaved differently in the GWR analysis. The Highland Park study area on the Brooklyn-Queens border exhibits a positive relationship between percent non-Hispanic black and physical activity site density, whereas the Marine Park study area in Brooklyn exhibits a negative association between the same two variables. Both parks have similar amounts of useable area, and possess a relatively high number of physical activity sites (see table 5). What might account for the differences reported in the GWR between the two study areas?

**Table 5:** Highland Park and Marine Park Comparison

	**Highland Park**	**Marine Park**
Park Construction (Year)	1901	1936

Park Size (Acres)	141	798*

Physical Activity (PA) Sites		
*Baseball Fields*	6	22

*Basketball Hoops*	8	21

*Golf Courses*	0	1

*Handball Courts*	10	22

*Playgrounds*	2	4

*Soccer Fields*	1	**

*Tennis Courts*	13	15

*Additional PA Sites*	none	bocce courts, cricket fields, hiking trails, skate park, kickball courts, and kayak/canoe launch sites

Capital Projects (money spent since 1995)	Over $6 million	Over $34 million

*Sample of Capital Projects*	New baseball field and tennis court lighting, added safety measures, synthetic turf soccer/football field (under construction), etc	Landscaping, construction of separate environmental and community centers, golf course irrigation, comfort stations, etc.

Quality of the Park	Good quality overall **(see figure 7e)**: well maintained older play equipment with safety surfaces **(see figure 7f)**, broken chess table (with caution tape), playground safety signs***	Good quality overall **(see figure 8c)**: some graffiti, play equipment with safety surfaces **(see figure 8d)**, "Clean up after yourself" signs, safety signs at every playground ***

Miscellaneous	Public School children maintain various gardens throughout the park **(figure 7d)**	n/a

*A large part of Marine Park is underwater, and this number includes an 18-hole golf course making Marine Park's usable area closer to the size of Highland Park. ** Baseball Fields double as soccer fields in Marine Park. ***Winter weather and construction projects seem to have temporarily reduced the overall quality of the parks, but this does not appear to be permanent.

An 800 m buffer, generally accepted as the upper end of "walking distance" [34], was drawn around the boundaries of each park to create the study areas. Census tracts that intersect this boundary were chosen for demographic analysis to represent the approximate catchment area of the park. The intersection was first performed by the GISc software, and the tracts which intersected but had a very small proportion of their area within the buffers were manually removed. For Highland Park, this area includes parts of the following neighborhoods: Highland Park and Cypress Hills, Brooklyn; and Glendale, Queens. For Marine Park, this area includes parts of the following neighborhoods: Gerritsen Beach, Sheepshead Bay, Marine Park, Flatlands, and Mill Island (all in Brooklyn).

### Highland Park

In 1891 Brooklyn purchased the land surrounding the Ridgewood Reservoir (built in 1856) to be used as a park. In 1905, the park was extended south, and by 1908 the park was extended west and set the boundaries that remain today. By 1908 the park already included football fields, baseball fields, tennis courts, several gardens, footpaths, and park structures. Situated among a chain of seventeen mid-19^th ^century cemeteries that straddle the Brooklyn-Queens border, Highland Park greenery blends in well with what is known in New York City as the "Cemetery Belt." [41]

**Figure 7:**
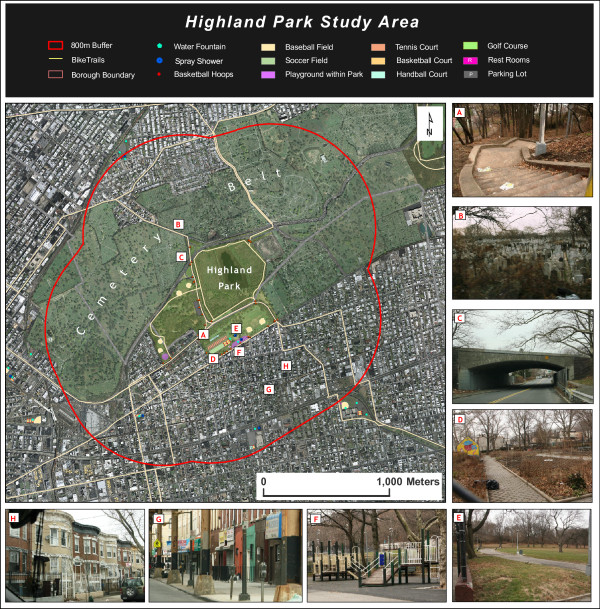
**a-h: Highland Park Study Area**. Data Sources: Orthophoto NYCMap, NYC Dept. of Information Technology and Telecommunication, 2002; NYC Dept. of Parks and Recreation collaboration with Lehman College "Geographic Features Identification Project," 2006; Photos by Kristen Grady, Lehman College Urban GISc Lab, 2009.

Highland Park's location is unique. A large portion of the park sits high atop the glacial moraine that runs diagonally from the southwest to the northeast through New York City, while the remainder of the park includes a steep slope that leads to a narrow piece of the park at the bottom of the moraine (see figure 7a). The Ridgewood Reservoir served as the impetus for creating the park. While there are a few baseball fields and a long bike path on the higher part of the park, most of the physical activity sites and playgrounds are located at the base of the park. The park's steep grade likely makes parts of the park difficult to access if you want to get to features on the other side. Even more problematic for park access are the less natural barriers that nearly cut the park off from Queens: a swath of cemeteries and the Jackie Robinson Parkway (see figure 7b and 7c), which both form the northern border of the park. There is only one access point to the park from Queens, which runs under the expressway via Cypress Avenue (see figure 7c). Park visitors entering from the north most likely do so by car, while those entering from the south have a much easier time accessing the park by walking. [41]

### Marine Park

Construction began on Marine Park in 1936 nearby undeveloped marshland around Gerritsen Creek. By 1937, the park included 1822 acres, in large part due to fill deposited in the marshes in the 1930s. With anticipated development, speculators purchased real estate along the waterfront. The vision of a new park inspired home building in the area which included a golf course built in 1963. In 1974, 1024 acres were transferred to the Gateway National Recreation Area. [42]

**Figure 8:**
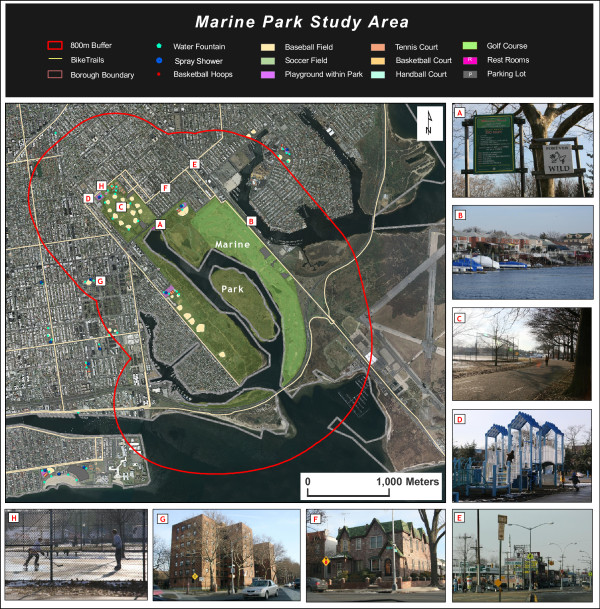
**a-h: Marine Park Study Area**. Data Sources: Orthophoto NYCMap, NYC Dept. of Information Technology and Telecommunication, 2002; NYC Dept. of Parks and Recreation collaboration with Lehman College "Geographic Features Identification Project," 2006; Photos by Kristen Grady, Lehman College Urban GISc Lab, 2009.

According to the New York City Department of Parks and Recreation, the park contains nearly 800 acres, although some of that acreage includes land that is under water. The park is adjacent to Rockaway Inlet and consists largely of salt marshes. The majority of the park's space is a protected "Forever Wild Preserve" (see figure 8a). Because the large 18-hole golf course fills the entire eastern portion of the park, the park's remaining physical activity sites are located in the north and southwestern regions of the park. Gerritson and Mill Creeks run north into the park, functionally separating the east and west sides from each other save for a sliver of greenery that connects the two sides in the north. Access to the park from the east is nearly impossible since a waterway (Mill Basin) borders the park here (see figure 8b). Access from the south is not possible either except for those who kayak in from Jamaica Bay via the Rockaway Inlet. Park access, then, is limited to the northern and western areas of the park, and not surprisingly, all physical activity sites, except for the golf course, are located in these areas. [42]

### Description of physical aspects of study areas

**Figure 9:**
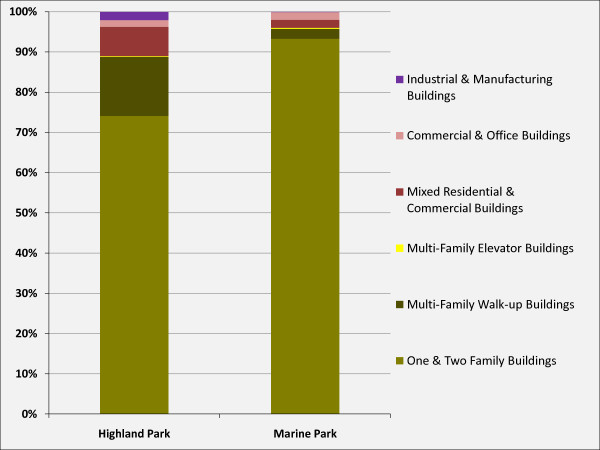
**Land-use Characteristics of the Two Study Areas, 2000**. Data Source: LotInfo, SpaceTrack, Inc.

Based on a GIS analysis of New York City land use data by property tax lot and ground-truthed by a visual inspection of the areas, both study areas were determined to be fairly similar in their land use makeup, with the vast majority of property lots containing residential buildings. However, the Highland Park study area consists of higher proportions of multi-family buildings, mixed commercial/residential, and industrial/manufacturing land uses whereas the Marine Park study area has a much higher proportion of one- and two-family buildings (see figure 9).

Both of the neighborhoods surrounding both Highland Park and Marine Park are heterogeneous in terms of levels of maintenance, upkeep, and cleanliness and there is a dramatic range of housing types, from mansions to low-income public housing complexes, found within the 800 m buffer of the parks, albeit not necessarily adjacent to one another (see figures 7h, 8f and 8g). There are also various types of business and commercial strips in both study areas (see figure 7g and 8e).

For both parks, it is difficult to qualitatively assess the association between the apparent socio-economic status of the neighborhood and accessibility to the park. For instance, the north side of Highland Park in Queens has much less access to the park than the comparatively less affluent neighborhood to the south of the park in Brooklyn. The Queens neighborhood is isolated from the park by obstacles such as cemeteries and a major highway (see figure 7b and 7c), and is by far the furthest neighborhood from the park's physical activity sites. One anomaly is that the mansions and larger homes located to the west of Highland Park appear to have excellent park access. It is interesting to note that when observing the Brooklyn side of the park alone, housing conditions do seem to deteriorate the further one travels away from the park.

For the Marine Park study area, park access appears more evenly distributed, regardless of SES. While the higher value housing around Marine Park is located near the main physical activity sites (see figure 8h), there is also high access to other physical activity sites from low-income housing complexes. There are some neighborhoods within the 800 m buffer of Marine Park that also have physical obstacles to access. The park itself contains natural areas of salt marsh and streams which are not easily traversable. There are also two neighborhoods in close proximity but separated from the park by water bodies (see figure 8b). One of these neighborhoods is a relatively higher income area and the other is lower-income.

### Socio-demographic characteristics of study areas

Since the construction of Highland Park and Marine Park, in 1891 and 1936, respectively, the demographics of the neighborhoods surrounding these two areas have changed considerably. Using the decennial censuses acquired from the National Historical Geographic Information System (NHGIS) [43], racial and ethnic categories were simplified to 'white,' 'black,' and 'other,' with Hispanic/Latino being considered 'other.' This was done to allow for longitudinal comparisons across the decades, since the US Census Bureau's categorization of racial and ethnic identity has been inconsistent over time.

**Figure 10:**
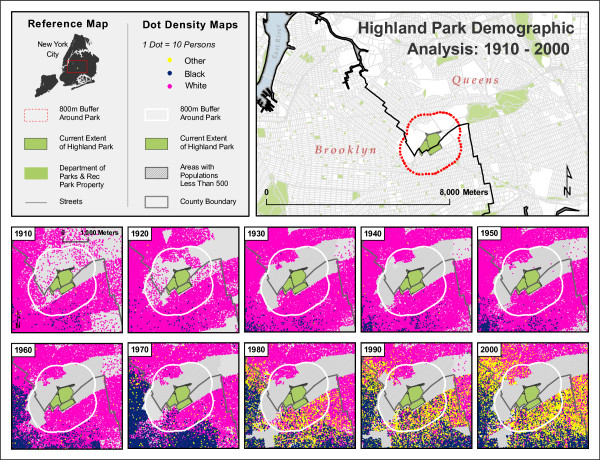
**Highland Park Demographic Analysis, 1910–2000**. Data Sources: US Bureau of the Census; National Historic Geographic Information System.

**Figure 11:**
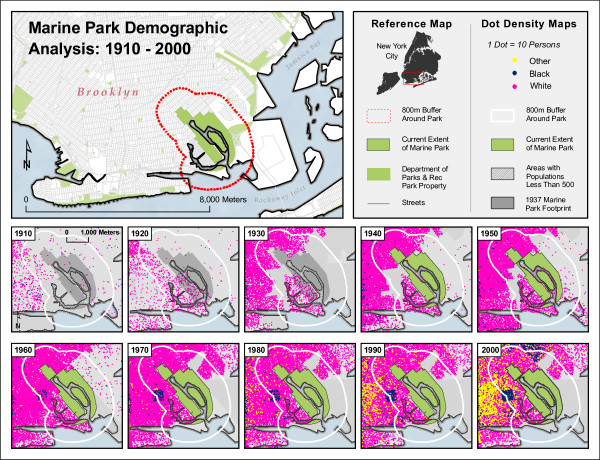
**Marine Park Demographic Analysis, 1910 – 2000**. Data Sources: US Bureau of the Census; National Historic Geographic Information System

From 1850 to 2000 both areas were transformed from rural farmland to dense urban areas. In the beginning of the 20^th ^century (1910 census), as shown in figures 10 and 11, both areas were occupied mainly by non-Hispanic white residents. The demographics of both neighborhoods remain relatively unchanged for five or six decades. The 1970 census shows a marked increase in the black population in the Highland Park area, while a similar change begins even earlier in the Marine Park area, although that area remains predominantly non-Hispanic white through the most recent census in 2000. The study area around Highland Park, however, experienced a huge transformation beginning in the decade between 1970 and 1980 with an influx of residents describing themselves as "other" and an increasing proportion of black population. This trend continues through the 2000 census.

**Figure 12:**
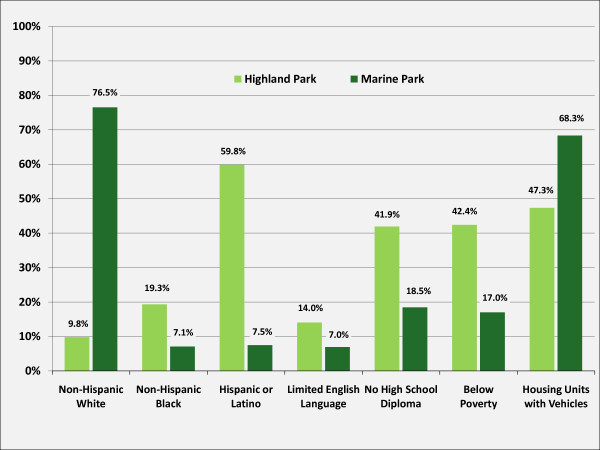
**Socio-demographic Characteristics of the Two Study Areas, 2000**. Data Source: U.S. Bureau of the Census, 2000.

A comparison of socio-demographic characteristics between the two study areas based on the more nuanced categories available in the 2000 census shows some differences (see figure 12). The Highland Park area is characterized by a high percentage of Hispanic population, a significant proportion of adult residents without a high school diploma, and a high poverty rate, whereas the Marine Park area is characterized by a predominantly non-Hispanic white population, lower poverty rates and a lower percentage of adults with no high school diploma. Furthermore, residents around Highland Park have a higher proportion of residents with limited ability to speak English than living near Marine Park. Both areas show a relatively high percentage of housing units with vehicles, however this percentage is higher in the Marine Park area than in the Highland Park area.

### Results of quantitative and qualitative analyses

Even though the OLS statistics suggest a trend toward racial/ethnic minorities and lower SES populations having higher access to parks and physical activity sites, the GWR points towards "unpatterned inequity," meaning that disparities in park access exist, but the inequity is inconsistently correlated with specific socio-demographic variables. This is further supported by the qualitative analysis, which implies that a global (city-wide) analysis of accessibility may not be the appropriate analytic method for this data. A limitation of the qualitative analysis is that only two case study park areas were examined, which is not likely to be representative of all the parks in New York City.

The variability of the quantitative and the qualitative results suggests a number of potential limitations and shortcomings of our analyses. These limitations are discussed in detail below.

## Limitations/future steps

As noted earlier in this paper, there are many pitfalls and problems in developing a meaningful park accessibility measure, and our analyses have reinforced the need for a more comprehensive approach. A number of additional variables might be included when measuring park access, in order to potentially establish more definitive results. In addition, our analysis focused on park access in New York City, which may or may not conform to the realities of other geographies.

### Proximity analysis based on "Walkability" distances

Access based on proximity or distance often does not take into account the actual street network, as noted earlier, and there may be a major highway or other barrier between the residents and the park. This was shown in our qualitative analysis of Highland Park, which is essentially cut off from the residential neighborhood on the northern side by large stretches of cemeteries and highways. Utilizing a network analysis may prove to be more realistic than the kernel estimation that we used, that quantified park access as a function of density of park acres and/or physical activities sites.

### Actual points of entry to the parks

A measure of access also needs to consider actual points of entry to the park. For some parks, there are entry points that may be at a far remove from the residential neighborhoods, even though the park may border the neighborhood. This can also be addressed using network analysis provided that the entry points are known and mapped.

### Perceptual access

Most accessibility measures do not take into account perceptual access. For example, racial/ethnic minority residents might not feel welcome in a park or recreational facility used predominantly by non-Hispanic white individuals. Similarly, female park users might not feel welcome in male-dominated physical activity sites such as basketball courts.

Perceptual access can also be based on park cleanliness or perceived or actual crime within or near the park. A park may be in close proximity but unused due to the bad conditions within the park. These "incivilities," such as graffiti, broken glass, liter, or evidence of drug or alcohol use, or the presence of violent crime could be important factors in making the park unattractive for use. While it is difficult to include perceptual access into a measure or index, if data on park maintenance and crime rates are available, they could be included in an analysis. Additionally, other more qualitative methods, such as interviews and surveys of local residents, as well as cognitive (mental) mapping and participatory GIS, can be used to discern attitudes and perceptions about access to parks and physical activity sites.

### Park characteristics

Most measures of accessibility do not consider park characteristics, either, such as types and quantities of activities available, or park size. A tiny vest-pocket park will not have as much to offer in the way of physical activity potential as a large park. Although our study did incorporate park acres and number of activity sites, it could be improved by looking at variation of the types of physical activity sites, (e.g., does the park contain only basketball courts, or are there tennis, basketball, and a variety of other types of sites within a given park?). Presumably, a park having a greater variety of types of physical activity sites would make the park more of a draw to residents of different ages, genders, and physical fitness status, and therefore that park would merit a higher rating.

Another park characteristic that may be taken into account is the requirement of permits in some physical activity sites. For instance, permits are required to use the tennis courts in all parks. Since these permits have fees, access is limited based on financial ability.

### Other variables to be considered

Resources available in parks and physical activity sites tend to be team sports-related, making it is less likely that older adults make use of these facilities. Therefore, the results of using physical activity sites as an important metric of accessibility may be misleading and skewed toward younger populations.

Additionally, people living in suburban-like areas within the city may have access to private open space, like backyards, usually available in single-family home neighborhoods, but not in higher-density, inner-city communities. Therefore proximity to public parks may be less important in those suburban-type areas, making any direct comparison of park and physical activity site accessibility between various types of neighborhoods and populations inaccurate. Public parks may serve a more critical function and there may be a higher need for public open space in less affluent neighborhoods, so statistical measures of equity regarding park access may not tell the whole story.

A valuable data source that was not explored in our qualitative analysis was comprehensive interviews with residents of the study areas to better understand actual and perceptual park accessibility. This could potentially provide important information necessary to address many of the limitations mentioned above.

### Census data

A major limitation of population studies such as this one is the necessarily heavy reliance on data from the census. While census data is the most complete and current dataset we have at any given time, we need to acknowledge several underlying problems with its accuracy. One of the most serious sources of inaccuracy is the potential for undercounting populations in poor and immigrant communities. This has been an on-going drawback throughout the United States, but is even more pronounced in 21^st ^century New York City, where a very high proportion of the population is foreign-born, and less likely to be counted in the census, especially in the case of illegal immigrants who may be mistrustful of government and wish to remain unknown to them. The temporarily or permanently homeless also comprise a significant population that is traditionally undercounted, as well as populations who may rotate their domicile and are therefore often overlooked in the official count because they are not thought of as being a permanent part of the respondent's household.

Additionally, each decennial census defines racial and ethnic categories differently, making cross-census comparisons difficult for longitudinal studies. The guidelines and standards for racial and ethnic classification were revised by the Office of Management and Budget in 1999, and the 2000 census uses a markedly different classification system from the previous censuses, making the findings of longitudinal studies somewhat unreliable.

### Policy implications

It is generally acknowledged that access to parks and physical activity sites has beneficial health ramifications, so a better understanding of which populations have good access to these areas will assist in identifying and targeting those areas that do not, and the potential for more fully explaining disparities in health outcomes. It is important to recognize the environmental justice implications of park and physical activity site location and spatial distribution, since ethnic and racial minorities and poor people tend to suffer disproportionately from diseases which are often preventable by proper exercise.

While we did not find an overall environmental justice impact for New York City as a whole with regard to park access and socio-demographic indicators, we know that there are many sections of the city with poor access to parks, and therefore this needs to be examined on a very local level rather than globally.

The level of need also has to be taken into account because even if parks were distributed evenly throughout the city, some neighborhoods warrant having additional resources. This may be due to the fact that these neighborhoods are more densely settled than other more suburban-like parts of the city, and their populations do not likely have additional open space resources such as backyards or options to leave the city for recreational opportunities. These are the very populations for whom parks assume an even more critical function than typically provided, and the parks and recreational facilities in these areas perhaps deserve extra resources and funding commitments in order to provide the equivalent level of support.

Physical, cultural, and perceptual barriers should be taken into account also when measuring access to parks. Even though distances may appear short, true access cannot be gauged through Euclidean measures, and much more research has to be done on what constitutes true access and equity of resources.

## Conclusion

The combination of quantitative and the qualitative analyses revealed trends that may not have been otherwise detectable. While the OLS (global) regression showed a weak relationship between SES characteristics and park accessibility, the geographically weighted regression (local) found "unpatterned inequality." The qualitative analysis did not reveal anything that would refute the statistical findings of the GWR analysis, as both neighborhoods were confirmed to be different from one another in terms of SES characteristics, although the park conditions and useable park area were comparable. The qualitative analysis did, however, suggest that an approach which considers physical barriers and some of the other variables listed in the limitation section would improve the model and better reflect reality. The qualitative analysis also showed that although the demographics around the study areas were similar at the times of the parks' construction, they have changed considerably since then, allowing for the possibility of environmental justice impacts. These environmental justice impacts may introduce disparities by influencing health outcomes and behaviors.

The complexity of the issues around racial and ethnic disparities in park access has been demonstrated further by this study. Looking at one factor at a time is likely to result in misleading findings. Therefore, a more complex model that accounts for as many different types of variables as possible (park size, access points, barriers, network distance, perception of safety, crime rates, park maintenance, availability and variation of physical activity sites) will be needed to develop a more accurate measurement of park accessibility, particularly as to how it might mediate environmental justice and mitigate negative health outcomes.

## Competing interests

The authors declare that they have no competing interests.

## Authors' contributions

AM designed and implemented the quantitative analysis, conducted exploratory data analysis and visualization of data, assembled and processed spatial datasets, created maps and diagrams, aided in the qualitative and longitudinal designs and analyses, and contributed to the writing and editing of the paper. JM conceived of the project concept, assisted in the development of the research design and analysis, supervised the quantitative and qualitative analyses, performed background research, and contributed to the writing and editing of the paper. NS helped with the data analysis and design, background research, and contributed to the writing of the paper. KG conducted the qualitative analysis, longitudinal demographic analysis, field work of case study areas, and archival research, and created map figures for the qualitative analysis. PA helped develop the project concept and contributed to writing and editing of paper. All of the authors have read and approved the final manuscript.
